# Dihydroaustrasulfone Alcohol (WA-25) Impedes Macrophage Foam Cell Formation by Regulating the Transforming Growth Factor-β1 Pathway

**DOI:** 10.3390/ijms160510507

**Published:** 2015-05-07

**Authors:** Yi-Chen Wang, Han-Chun Hung, Chien-Wei Feng, Shi-Ying Huang, Chun-Hong Chen, Yen-You Lin, Yao-Chang Chen, San-Nan Yang, Jui-Hsin Su, Jyh-Horng Sheu, Zhi-Hong Wen

**Affiliations:** 1 Department of Marine Biotechnology and Resources, National Sun Yat-sen University, Kaohsiung 80424, Taiwan; E-Mails: cvyc.wang@gmail.com (Y.-C.W.); chas6119@gmail.com (Y.-Y.L.); 2 Division of Cardiology, Department of Internal Medicine, Kaohsiung Armed Forces General Hospital, Kaohsiung 80284, Taiwan; 3 Doctoral Degree Program in Marine Biotechnology, National Sun Yat-sen University and Academia Sinica, Kaohsiung 80424, Taiwan; E-Mails: hanchun25@gmail.com (H.-C.H.); qscjuejuejue@gmail.com (C.-W.F.); anubis0620@gmail.com (C.-H.C.); 4 Center for Neuroscience, National Sun Yat-sen University, Kaohsiung 80424, Taiwan; E-Mail: johnjohnkings@gmail.com; 5 Department of Biomedical Engineering, National Defense Medical Center, Taipei 11490, Taiwan; E-Mail: bme02@mail.ndmctsgh.edu.tw; 6 School of Medicine, College of Medicine and Department of Pediatrics, E-DA Hospital, I-Shou University, Kaohsiung 84001, Taiwan; E-Mail: y520729@gmail.com; 7 National Museum of Marine Biology and Aquarium, Pingtung 94450, Taiwan; E-Mail: x2219@nmmba.gov.tw; 8 Graduate Institute of Natural Products, Kaohsiung Medical University, Kaohsiung 80708, Taiwan; 9 Department of Medical Research, China Medical University Hospital, China Medical University, Taichung 40402, Taiwan; 10 Marine Biomedical Laboratory & Center for Translational Biopharmaceuticals, Department of Marine Biotechnology and Resources, National Sun Yat-sen University, Kaohsiung 80424, Taiwan

**Keywords:** marine compounds, macrophage, foam cell, lysosome, cyclic adenosine monophosphate (cAMP), transforming growth factor β1 (TGF-β1)

## Abstract

Atherosclerosis is considered an inflammatory disease. However, clinically used anti-atherosclerotic drugs, such as simvastatin, have many side effects. Recently, several unique marine compounds have been isolated that possess a variety of bioactivities. In a previous study, we found a synthetic precursor of the marine compound (austrasulfone), which is dihydroaustrasulfone alcohol (WA-25), has anti-atherosclerotic effects *in vivo*. However, the detailed mechanisms remain unclear. Therefore, to clarify the mechanisms through which WA-25 exerts anti-atherosclerotic activity, we used RAW 264.7 macrophages as an *in vitro* model to evaluate the effects of WA-25. In lipopolysaccharide (LPS)-stimulated RAW 264.7 cells, WA-25 significantly inhibited expression of the pro-inflammatory proteins, inducible nitric oxide synthase (iNOS) and cyclooxygenase-2 (COX-2). In contrast, simvastatin increased the COX-2 expression compared to WA-25. In addition, WA-25 impedes foam cell formation and up-regulated the lysosomal and cyclic adenosine monophosphate (cAMP) signaling pathway. We also observed that transforming growth factor β1 (TGF-β1) was up-regulated by WA-25 and simvastatin in LPS-induced RAW 264.7 cells, and the promising anti-atherosclerosis effects of WA-25 were disrupted by blockade of TGF-β1 signaling. Besides, WA-25 might act through increasing lipolysis than through alteration of lipid export. Taken together, these data demonstrate that WA-25 may have potential as an anti-atherosclerotic drug with anti-inflammatory effects.

## 1. Introduction

According to statistics published by the World Health Organization, more than 17.3 million people suffer from cardiovascular diseases (CVDs) in industrialized societies, and this number will increase to 23.3 million by 2030 [1]. Among the CVDs, atherosclerosis is the leading cause of mortality and morbidity worldwide in a variety of populations, and it is responsible for substantial health and social burdens [2]. Atherosclerosis is a multifactorial diseases characterized and maintained by abnormal lipid metabolism [3]. There are many risk factors associated with atherosclerosis, including dyslipidemia, hypertension, and smoking. However, recent studies indicate that inflammation is another major cause of atherosclerosis [4,5] and that it plays an important role in the formation of atherosclerotic plaques [6]. In the initiation phase of plaque development, fatty streak formation and macrophage accumulation are observed [7], and macrophages and pro-inflammatory molecules are found in symptomatic plaques [8]. Moreover, inflammation plays a role in plaque rupture and destabilization [9]. Therefore, the detailed mechanisms of inflammation in macrophages associated with atherosclerosis should be clarified.

Statins are usually regarded as the first line therapy for atherosclerosis. Statins are 3-hydroxy-3-methylglutaryl-coenzyme A (HMG-CoA) reductase inhibitors that lower cholesterol levels [10]. In clinical studies, statins have been reported to reduce the risk of atherosclerosis and regulate lipid levels [11,12]. However, some statins have serious side effects such as neuropathy, hepatic dysfunction, sexual dysfunction and even rhabdomyolysis [13,14]. Therefore, drug discovery efforts aimed at atherosclerosis therapies must carefully evaluate drug safety and further clarify the detailed molecular mechanisms of atherosclerosis pathogenesis. Because of the limitations of current atherosclerosis therapies, it is imperative that alternative treatment strategies be developed.

Oceans cover more than 70% of the surface of the Earth, and they have extremely high species diversity [15]. Because of the diversity of oceanic flora and fauna, marine compounds represent new sources of drugs that could be used to treat atherosclerosis. Marine compounds have been shown to have diverse bioactivities, such as anti-inflammatory, anti-neuroinflammatory, and neuroprotective effects [16,17]. In addition, some marine compounds have the potential to prevent and treat cardiovascular disease [18]. Our previous results indicated that austrasulfone extracted from the Formosan soft coral *Cladiella australis* has several bioactivities [19]. Importantly, we developed a simple method to synthesize austrasulfone using a 2-step reaction. Dihydroaustrasulfone alcohol (WA-25), the austrasulfone precursor used in our synthesis, possesses anti-inflammatory activity and therapeutic effects against neuropathic pain, atherosclerosis, multiple sclerosis, and cancer [19,20]. Although studies show that marine-derived compound (WA-25) may represent a potential therapeutic agent for atherosclerosis, the detailed mechanisms through which WA-25 exerts anti-atherosclerosis effects are unknown.

Early in the development of atherosclerosis, lipid droplets usually accumulate in macrophages and cause formation of foam cells [21]. When foam cells accumulate in the arterial wall, they cause fatty streaks [22,23]. Therefore, foam cells are considered a major cause of atherosclerosis. Macrophages transform into foam cells because of the accumulation of excess lipid bodies. Lipogenesis and expression of adipophilin can be observed in macrophages *in vitro* [24]. In addition, some studies indicate that macrophages involved in the process of atherosclerosis up-regulate protein and mRNA levels of inducible nitric oxide synthase (iNOS) and cyclooxygenase-2 (COX-2) [25]. In contrast, the lysosomal system of macrophages has been shown to mediate anti-atherogenic effects. These results suggest that inflammatory macrophages play a critical role in lipid metabolism. In addition, cyclic adenosine monophosphate (cAMP) also plays an important role in lipolysis [26]. Catecholamines are examples of substances that improve lipolysis by increasing intracellular cAMP [27]. Lipopolysaccharides (LPS) can cause macrophages to secrete pro-inflammatory mediators such as iNOS and COX-2 [28]. The RAW 264.7 cell line is murine macrophage cell line that can be used for evaluating the anti-inflammatory effects of test compounds [29]. Because inflammation has recently been shown to have a role in the development of atherosclerosis, RAW 264.7 cells are considered a valid *in vitro* model with which to study atherosclerosis. RAW 264.7 cells have been used in many lipogenesis and lipolysis experiments [30]. RAW 264.7 cells can be loaded with oleic acid (OA) to contribute to lipid droplet formation, and lipid droplets in macrophages can be detected via Nile red fluorescence [31]. Therefore, Nile red staining is used to evaluate lipid droplet metabolism in RAW 264.7 cells. Macrophages can be activated by oxidized LDL (oxLDL) in the early stages of atherosclerosis development. Several studies indicated that CD36 plays an important role in oxLDL recognition by macrophages [32]. However, the role of CD36 has recently been questioned because it might make atherosclerosis worse if treatment improve the lipolysis but reduces the CD36-mediating export of lipid. Therefore, it is important to determine the expression of CD36 when analyzing new drugs for the treatment of atherosclerosis.

Numerous studies have shown that atherosclerosis is a chronic inflammation resulting from dysregulation between many factors such as macrophages, T cells and lipoproteins [33]. However, the mechanism of inflammation is quite complex. Among them, transforming growth factor (TGF)-β plays a crucial role in regulating adaptive and inflammatory immune responses [34]. TGF-β family has three isoforms, among which TGF-β1 is regarded as an anti-inflammatory cytokine [35]. Recently, Lee and colleagues suggested that TGF-β1 could alleviate inflammation by inhibiting toll-like receptor (TLR)-mediated signaling [36]. However, the mechanisms through which TGF-β1 signaling might regulate atherosclerosis remain unclear. Therefore, TGF-β1-related signaling should be further investigated in the context of atherosclerosis.

In this study, we examined the detailed anti-atherosclerotic mechanisms of marine compound (WA-25). First, we utilized RAW 264.7 cells as an *in vitro* model to evaluate the anti-inflammatory activity of WA-25 and its effect on lipid droplet metabolism in comparison with simvastatin. Next, we investigated the effects of WA-25 on the expression of lysosomal-associated membrane protein 1 (LAMP-1), cAMP and TGF-β1. In brief, we attempt to clarify the detailed mechanisms of WA-25.

## 2. Results

### 2.1. Dihydroaustrasulfone Alcohol (WA-25) Suppresses the Inducible Nitric Oxide Synthase (iNOS) and Cyclooxygenase (COX)-2 Protein Expression in Lipopolysaccharide (LPS)-Induced RAW 264.7 Cells

**Figure 1:**
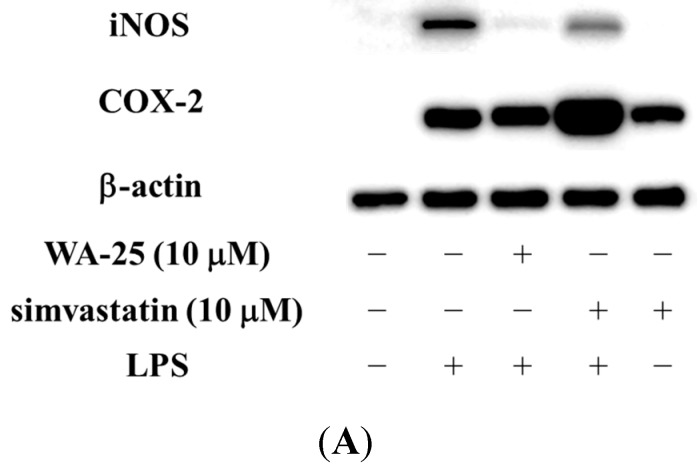

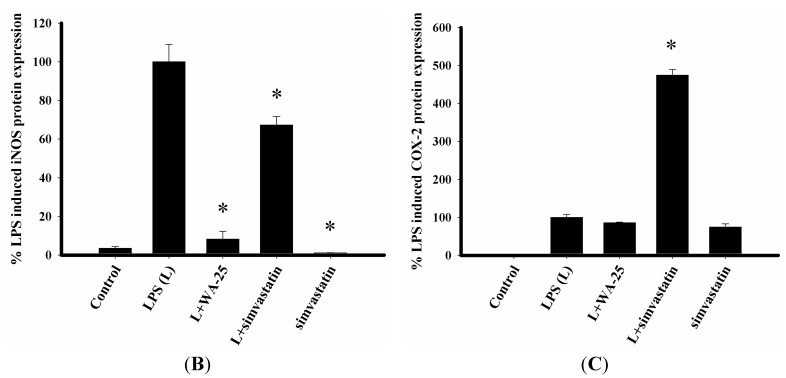
Effects of dihydroaustrasulfone alcohol (WA-25) on expression of inducible nitric oxide synthase (iNOS) and cyclooxygenase (COX)-2 proteins in RAW 264.7 macrophage cells, induced by lipopolysaccharide (LPS) for 16 h. (**A**) Western blots for iNOS, COX-2 and β-actin proteins from RAW 264.7 macrophage cells; (**B**) Relative immunointensity of iNOS; (**C**) Relative immunointensity of COX-2. The relative intensity of the LPS-stimulated group was taken to be 100%. Band intensities were quantified by densitometry and are indicated as a percentage change relative to that of the LPS-stimulated group. Western blotting with β-actin was performed to verify that equivalent amounts of protein were loaded in each lane. This experiment was repeated three times with similar observation. *, significantly different from the LPS-stimulated group (*p* < 0.05).

To determine whether WA-25 suppressed iNOS and COX-2 protein expression, cells were stimulated with LPS and expression of pro-inflammatory proteins was measured by Western blot. Simvastatin was used as a positive control. Therefore, RAW 264.7 cells were treated with WA-25 or simvastatin and then stimulated with LPS for 16 h. As shown in Figure 1A,B, 10 μM WA-25 significantly down-regulated LPS-induced expression of iNOS more effectively than simvastatin. In addition, 10 μM WA-25 decreased COX-2 expression but not significantly (Figure 1A,C). In contrast, we found that simvastatin up-regulated COX-2 in unstimulated RAW 264.7 cells. The inhibitory effects of WA-25 were not a result of cytotoxic effects. These data indicate that WA-25 might be effective as an anti-inflammatory agent. However, we also found that simvastatin might activate inflammatory signaling pathway in macrophages.

### 2.2. Effect of WA-25 on Lipolysis in Lipid-Laden Macrophages

**Figure 2:**
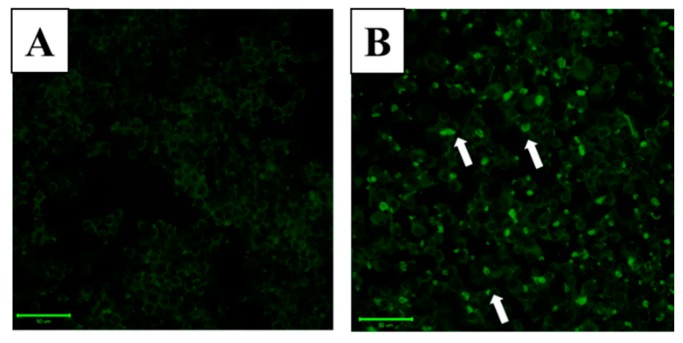

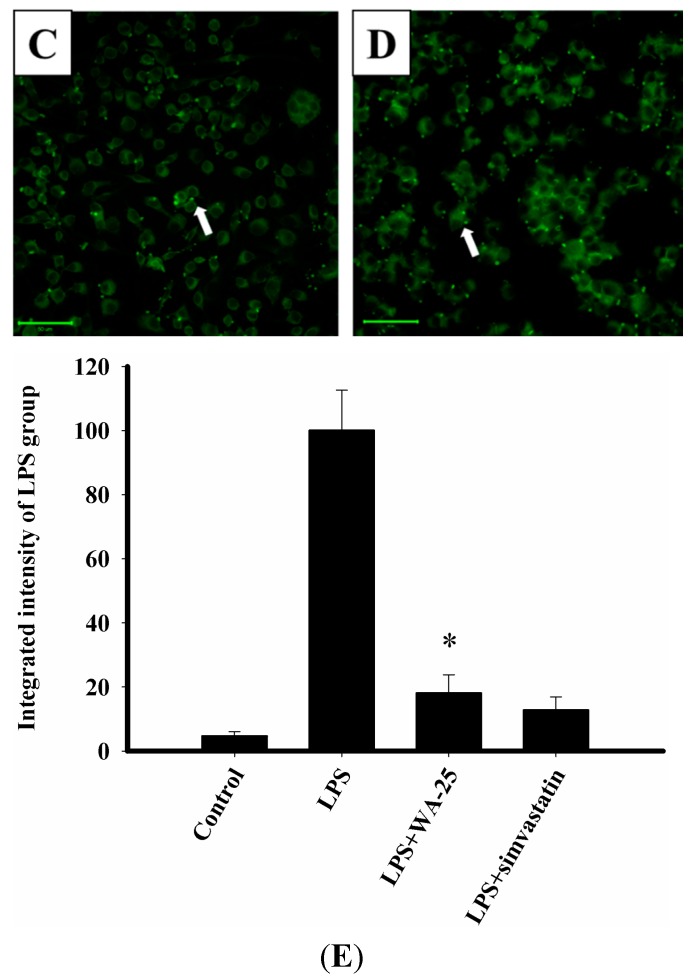
Nile red staining of lipid droplets in RAW 264.7 macrophages. Cells were loaded with (**A**) Dimethyl sulfoxide (DMSO); (**B**) LPS; (**C**) LPS and WA-25 (10 μM); (**D**) LPS and simvastatin (10 μM) in the presence of oleic acid (OA) for 16 h. The arrows indicate lipid droplets; (**E**) The integrated intensity of immunofluorescent staining of lipid droplet. The number of lipid droplets was significantly decreased after WA-25 and simvastatin treatment. Scale bar, 50 μm. These experiments were repeated three times, and images of at least six different fields were taken for analysis. *, significantly different from the LPS-stimulated group (*p* < 0.05).

In order to investigate the effect of WA-25 on lipolysis in lipid-laden macrophages, we used the lipid droplet assay with Nile red staining to stain the droplets. LPS dramatically increased lipid droplet accumulation in RAW 264.7 cells in comparison with control cells (Figure 2A,B). Moreover, we found that 10 μM WA-25 and simvastatin notably decreased LPS-triggered macrophage foam cell formation (Figure 2C,D). Besides, the integrated density showed the same results as immunofluorescence staining (Figure 2E). These results showed that both WA-25 and simvastatin improve lipolysis in lipid droplets.

### 2.3. LAMP-1 and cAMP Involvement in the Effect of WA-25 on Lipolysis

**Figure 3:**
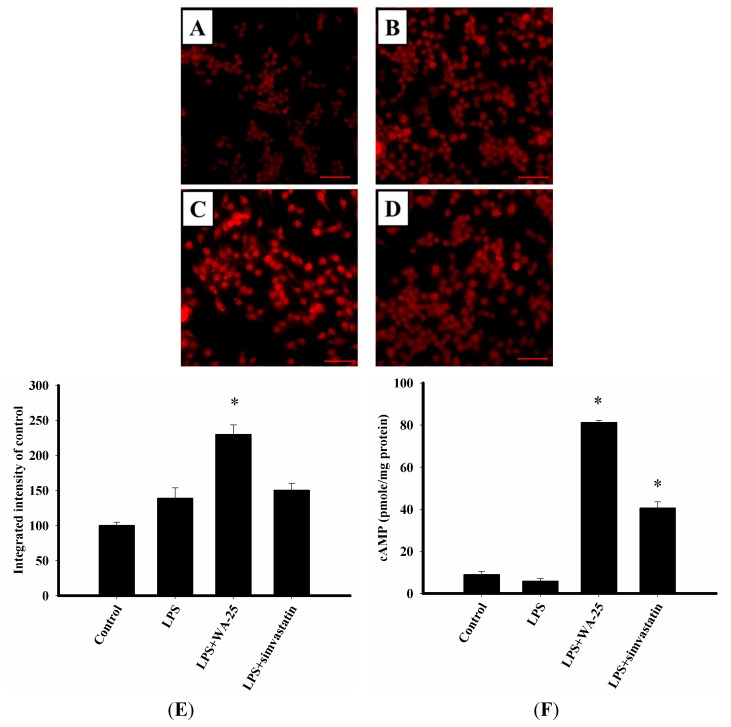
Effects of WA-25 and simvastatin on the expression of lysosome and cAMP level in RAW 264.7 murine macrophages induced by LPS. Cells were immunostained with anti-LAMP1 proteins. RAW 264.7 were incubated for 16 h with (**A**) the DMSO control; (**B**) LPS; (**C**) LPS + WA-25 (10 μM); or (**D**) LPS + simvastatin (10 μM); (**E**) The integrated intensity of immunofluorescent staining of LAMP-1 protein; (**F**) The cAMP level. Scale bar, 50 μm. These experiments were repeated three times, and images of at least six different fields were taken for analysis. *, significantly different from the LPS-stimulated group (*p* < 0.05).

To investigate whether WA-25 induced lipolysis by enhancing the function of the lysosome system, we carried out immunofluorescence staining to visualize changes in protein expression of the lysosome marker LAMP-1. As shown in Figure 3A, unstimulated RAW 264.7 cells expressed low levels of LAMP-1 protein. LPS slightly increased LAMP-1 expression, whereas WA-25 remarkably increased LAMP-1 expression, and simvastatin did not change LAMP-1 expression (Figure 3B–D). Simultaneously, the integrated density showed the same results as immunofluorescence staining (Figure 3E). In addition, we examined whether WA-25 enhanced lipolysis by regulating the cAMP-dependent pathway. As shown in Figure 3F, WA-25 increased cAMP levels significantly compared to the LPS group and more effectively than simvastatin. Collectively, the data showed that WA-25 might induce lipolysis that appears to be mediated by lysosome-related and cAMP-related mechanisms.

### 2.4. WA-25 Disrupts LPS-Induced Down-Regulation of TGF-β1 Protein

**Figure 4:**
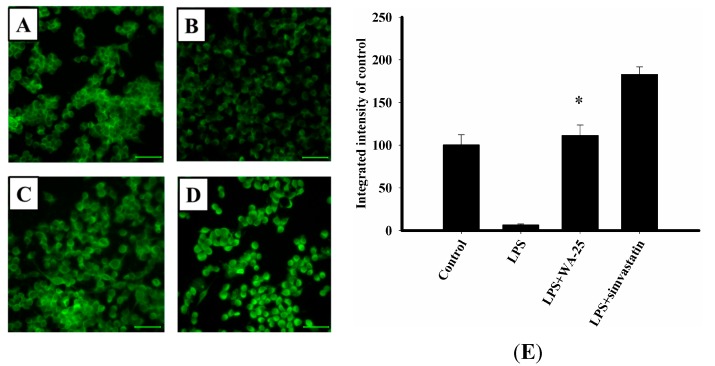
Effects of WA-25 or simvastatin on the expression of TGF-β1 in RAW 264.7 murine macrophages induced by LPS. Cells were immunostained with anti TGF-β1 proteins. RAW 264.7 were incubated for 16 h with (**A**) the DMSO control; (**B**) LPS; (**C**) LPS + WA-25 (10 μM); or (**D**) LPS and simvastatin (10 μM); (**E**) The integrated intensity of immunofluorescent staining of TGF-β1 protein. Scale bar, 50 μm. These experiments were repeated three times, and images of at least 6 different fields were taken for analysis. *, significantly different from the LPS-stimulated group (*p* < 0.05).

To determine the detailed molecular mechanisms of WA-25, we examined TGF-β1 expression by immunocytochemistry. RAW 264.7 cells were treated with WA-25 or simvastatin and then stimulated with LPS. After treatment, we found that LPS significantly down-regulated TGF-β1 expression in comparison with the unstimulated group (Figure 4A,B). As shown in Figure 4C, WA-25 restored TGF-β1 expression that was reduced by LPS. Interestingly, we also observed that simvastatin increased TGF-β1 expression as effectively as WA-25 (Figure 4D). Besides, the integrated density showed the same results (Figure 4E). Importantly, these results showed that TGF-β1 might play an important role in the anti-atherosclerotic effects of WA-25 and simvastatin.

### 2.5. The Effect of TGF-β1 Inhibitor (SB-431542) on the Anti-Inflammatory Effect of WA-25

**Figure 5:**
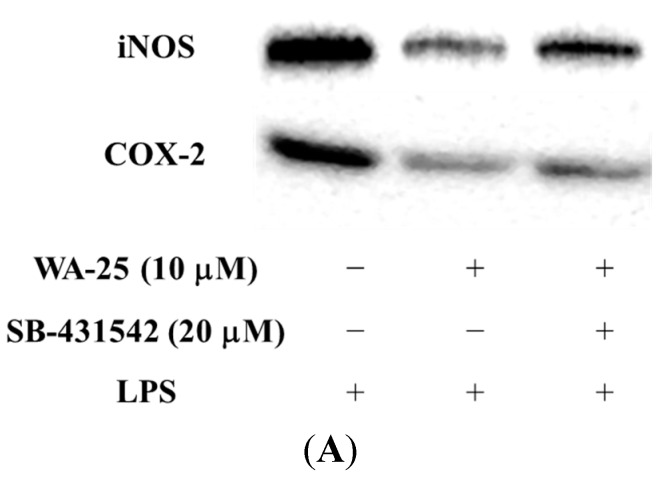

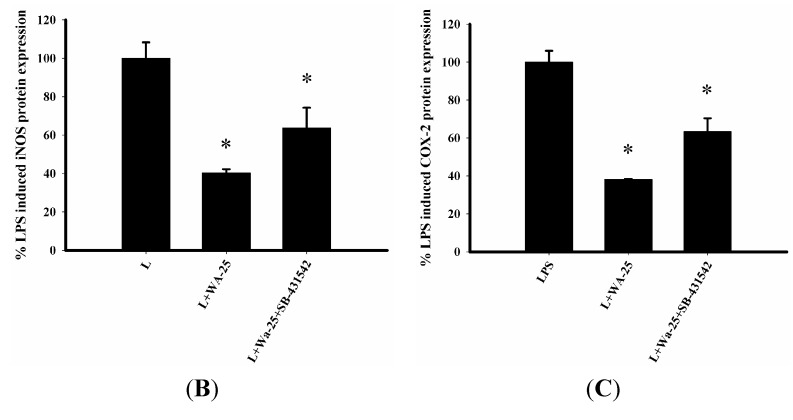
SB-431542 (TGF-β1 inhibitor) blocks the down-regulation of iNOS and COX-2 by WA-25 in RAW 264.7 macrophage cells induced by LPS for 16 h (**A**); (**B**) Western blots for iNOS and the histogram showed the relative intensity; (**C**) Western blots for COX-2 and the histogram showed the relative intensity. This experiment was repeated three times, with similar observation in each experiment. *, significantly different from the LPS-stimulated group (*p* < 0.05).

To examine whether TGF-β1 is associated with the ability of WA-25 to inhibit inflammation and induce lipolysis, we exposed RAW 264.7 cells to TGF-β1 receptor inhibitor SB-431542 and measured protein expression by Western blot. As shown in Figure 5A, down-regulation of iNOS by WA-25 was significantly disrupted by SB-431542. In addition, SB-431542 blocked the inhibitory effect of WA-25 on LPS-induced COX-2 expression (Figure 5B). Based on these results, we speculate that the anti-inflammatory effect of WA-25 may be mediated by TGF-β1-related signaling.

**Figure 6:**
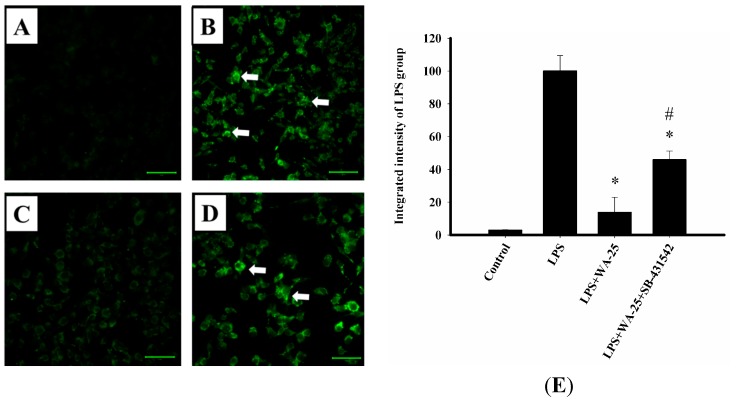
SB-431542 (TGF-β1 inhibitor) blocks the lipolytic effect of WA-25 in RAW 264.7 macrophages stimulated by LPS. Cells were loaded with (**A**) DMSO; (**B**) LPS; (**C**) LPS + WA-25 (10 μM); or (**D**) LPS + WA-25 (10 μM) + SB-431542 (20 μM) in the presence of OA for 16 h. The arrows indicate lipid droplets; (**E**) The integrated intensity of immunofluorescent staining of lipid droplet. Scale bar, 50 μm. These experiments were repeated three times, and images of at least six different fields were taken for analysis. *, significantly different from the LPS-stimulated group (*p* < 0.05). #, significantly different from the LPS + WA-25 (10 μM) group (*p* < 0.05).

### 2.6. TGF-β1 Inhibition Blocked the Lipolytic Effect of WA-25 in RAW 264.7 Cells

To further confirm our findings, we examined whether TGF-β1 inhibitor SB-431542 blocked lipolysis induction by WA-25. LPS dramatically increased lipid droplet accumulation in RAW 264.7 cells in comparison with control cells (Figure 6A,B). As shown in Figure 6C, treatment with WA-25 significantly reduced lipid droplet abundance. However, TGF-β1 inhibitor SB-431542 blocked the lipolytic effect of WA-25 (Figure 6D). In addition, the integrated density showed the same results as immunofluorescence staining (Figure 6E). Taken together, our data suggest that WA-25 could regulate inflammation and lipolysis in LPS-treated macrophages by enhancing TGF-β1 signaling.

### 2.7. WA-25 Does not Change Alter CD-36 Expression, But it Can Decreases the Accumulation of Oxidized LDL (oxLDL)

**Figure 7:**
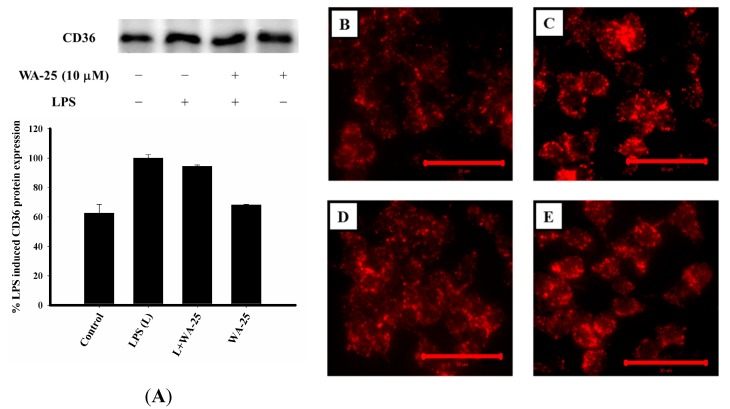
Effects of WA-25 on CD36 expression and LDL uptake in LPS-induced RAW 264.7 murine macrophages. (**A**) Western blots for CD36 and histogram showing the relative intensity; Cells were treated with LDL conjugated to DyLight™ 549 and incubated for 16  h with (**B**) DMSO (control); (**C**) LPS; (**D**) LPS + WA-25 (10 μM); or (**E**) WA-25 (10 μM). Scale bar, 30 μm. These experiments were repeated three times, and images of at least six different fields were taken for analysis.

To determine whether WA-25 could affect lipid uptake, we examined the protein expression of CD36, which mediates oxLDL uptake. As shown in Figure 7A, LPS treatment increased CD36 expression. In contrast, WA-25 treatment did not change CD36 expression markedly. In addition, we also examined LDL uptake into RAW 264.7 cells using LDL conjugated to DyLight™ 549 as a fluorescent probe. As shown in Figure 7B–E, oxLDL uptake was significantly lower in the LPS + WA-25 group than in the LPS group. In addition, WA-25 alone group did not increase CD36 expression. Collectively, these data show that WA-25 likely acts by improving lipolysis than by altering the export of lipids.

## 3. Discussion

In the present study, we used a RAW 264.7 macrophage cell *in vitro* model of LPS-stimulated inflammation to evaluate the anti-atherogenic effect of WA-25. Based on the importance of inflammation and lipid metabolism in atherosclerosis, we focused on assessing the anti-inflammatory and lipolytic effects of WA-25. In the Western blot analysis, we found that 10 μM WA-25 reduced protein expression of iNOS and COX-2, whereas simvastatin reduced iNOS expression, but increased COX-2 expression. Nile red staining to examine lipolysis induction by WA-25 showed that, surprisingly, WA-25 inhibited LPS-induced accumulation of lipid droplets in RAW 264.7 macrophages. We also found that the anti-inflammatory and pro-lipolysis effects of WA-25 might be mediated by signaling involving LAMP-1, cAMP and TGF-β1.

Atherosclerosis is quite common in cardiovascular disease and is the leading cause of death worldwide [37]. Despite the availability of prescription drugs to combat heart disease, many patients suffer from major adverse cardiovascular events. The continued prevalence of atherosclerosis may be caused by the lack of focus of current therapies on inflammation [38]. Recent reports suggest that inflammation might play a primary role in atherosclerosis [39]. Simultaneously, numerous studies have reported that iNOS and COX-2 play an important role in inflammation [40]. Statins are usually used to treat atherosclerosis, and recent studies demonstrate that statins also have anti-inflammatory effects [41]. However, statins have common side effects that limit their use. Based on above, we try to find new anti-inflammatory compound to be used in atherosclerosis. Our previous studies suggested that the bioactive substance WA-25 from the Formosan soft coral *Cladiella australis* might have multiple therapeutic uses for treatment of neuropathic pain, atherosclerosis, multiple sclerosis, and lung cancer [19]. Despite these promising results, we could not clarify the signaling pathway through which the therapeutic effects of WA-25 in atherosclerosis are mediated. In addition, we wondered whether WA-25 could be more effective than simvastatin as a clinical treatment for atherosclerosis. In this study, we used RAW 264.7 macrophage cells as an *in vitro* model to evaluate the effects of WA-25 and simvastatin. WA-25 inhibited LPS-stimulated iNOS and COX-2 protein expression. Interestingly, simvastatin inhibited iNOS expression in LPS-stimulated cells, and enhanced COX-2 expression in unstimulated cells. These results were not been reported in previous studies and suggested that simvastatin may have produce side effects via up-regulation of COX-2. These results demonstrate that WA-25 could be more effective than simvastatin as an anti-inflammatory agent.

Accumulation of lipid droplets in macrophages causes formation of foam cells [42]. The macrophages express iNOS and COX-2 could be found in atherosclerotic lesions [25]. Therefore, inflammation may reduce macrophage lipolysis. In the present study, we used a lipid droplet assay to assess lipolysis induced by WA-25. Our results indicated that WA-25 might enhance lipolysis in LPS-treated RAW 264.7 macrophage cells. Simvastatin also decreased the accumulation of lipid droplets, and it was more effective than WA-25. However, a recent study indicated that simvastatin induced lipid droplet accumulation in a manner that caused cell death [43]. Therefore, there are risks associated with the use of simvastatin as a treatment for atherosclerosis. These results suggest that WA-25 may improve lipid metabolism.

LAMP-1 is a major constituent of the lysosomal membrane that can be used as a lysosome marker. Lysosomes are able to break down lipids, organelles, and proteins [44], and recently lysosome have been shown to play a critical role in the early stage of atherosclerosis. In addition, reduced expression of LAMP-1 has been observed in atherosclerosis [45]. Emanuel *et al.* demonstrated that induction of lysosomal activity in macrophages has anti-atherogenic effects [46]. The cAMP-dependent protein kinase (PKA) signaling pathway activates the expression of hormone-sensitive lipase (HSL), which improves lipolysis [47]. These results suggest that WA-25 up-regulates the expression of LAMP-1 and elevates the cAMP levels in LPS-induced RAW 264.7 macrophage cells. Collectively, these observations confirm that WA-25 may enhance lipolysis by up-regulating lysosomal activity and the cAMP-PKA pathway.

TGF-β1 is secreted from many cell types, including macrophages, endothelial cells, and T cells [36]. Evidence from animal studies indicates that TGF-β1 is an anti-atherogenic cytokine [48]. Many drugs stimulate TGF-β1 expression, including HMG-CoA reductase inhibitors such as statins [49]. Interestingly, our data showed that WA-25 and simvastatin reversed LPS-induced decreases in TGF-β1 levels. Based on these results, we speculated that TGF-β1 might play an important role in the anti-atherogenic effects of WA-25 and simvastatin. In order to examine whether TGF-β1 was involved in the effect of WA-25, we used the TGF-β1 inhibitor SB-431542 to block TGF-β1 signaling. Our results indicated that down-regulation of iNOS through the anti-inflammatory effect of WA-25 was significantly inhibited by SB-431542. Simultaneously, we also found that down-regulation of COX-2 by LPS was also disrupted significantly by WA-25. We used SB-431542 to examine whether it affected the ability of WA-25 to induce lipolysis. As shown in Figure 6, we found that SB-431542 blocked promotion of lipolysis by WA-25. Therefore, we suggest that WA-25 may inhibit inflammation and induce lipolysis through TGF-β1 signaling.

CD36 is a member of the scavenger receptor B family. It plays an important role in atherosclerosis progression and the formation of foam cells [50]. Cells contain many CD36 ligands, including oxLDL and LPS. A recent study indicated that abnormal lipid accumulation is affected by CD36 [51]. However, another study demonstrated that cold-triggered food-intake–independent lipolysis significantly increased plasma levels of LDL and accelerates the development of atherosclerosis [52]. Therefore, if treatment increases lipolysis but reduces the CD36-mediating export of lipid, it might aggravate atherosclerosis. Our results show that CD36 expression in the LPS group was higher than that in the control group (Figure 7A). In contrast, the WA-25 treatment group did not show any marked change in the LPS-induced up-regulation of CD36. We further examined the effect of WA-25 on oxLDL uptake. As shown in Figure 7B–E, the LPS group showed abundant accumulation of oxLDL in RAW 264.7 cells. However, WA-25 treatment decreased oxLDL accumulation. These results suggest that WA-25 might act through increasing lipolysis than through alteration of lipid export.

## 4. Experimental Section

### 4.1. Chemicals

Dihydroaustrasulfone alcohol (WA-25) was partly provided by Jyh-Horng Sheu, Department of Marine Biotechnology and Resources of National Sun Yat-sen University and research center of National Research Program for biopharmaceuticals, Taiwan (http://nrpb.sinica.edu.tw/zh-hant/rc). Penicillin, streptomycin, dimethyl sulfoxide (DMSO), lipopolysaccharide (LPS) from *Escherichia coli*, simvastatin, anti-β-actin antibody and SB-431542 were obtained from Sigma-Aldrich Chemical Co. (St. Louis, MO, USA). Dulbecco’s modified Eagle’s medium (DMEM), fetal bovine serum (FBS), sodium pyruvate, l-glutamine, penicillin-streptomycin, and trypsin-EDTA were purchased from Invitrogen Co. (Grand Island, NY, USA). The anti-iNOS and COX-2 antibody and lipid droplets fluorescence assay kit and LDL uptake cell-based assay kit were purchased from Cayman Chemical Company (Ann Arbor, MI, USA). The anti-LAMP-1 antibody was obtained from Millipore Corp (Billerica, MA, USA). The anti-TGF-β1 and CD36 were purchased from Abcam Corp (Cambridge, UK). The horseradish peroxidase-conjugated secondary antibody, Alexa Fluor 488 (green fluorescence) or 594 (red fluorescence)-conjugated secondary antibody were purchased from Jackson ImmunoResearch Laboratories (West Grove, PA, USA).

### 4.2. RAW 264.7 Macrophage Cell Line Culture

The RAW 264.7 macrophage cell line was cultured as described previously [16,53]. RAW 264.7 macrophages (No. TIB-71) were obtained from the American Type Culture Collection (ATCC, Manassas, VA, USA) and cultured in DMEM containing 10% heat-inactivated FBS, 2 mM glutamine, 1 mM pyruvate, 4.5 g/L glucose, 50 U/mL penicillin, and 50 μg/mL streptomycin. The RAW 264.7 cultures were maintained at 37 °C in a humidified atmosphere containing 5% CO_2_ and 95% air. RAW 264.7 cells were trypsinized for subculture in different-sized wells or dishes. Culture plasticware was obtained from Corning Inc. (Corning, NY, USA). All experiments were performed overnight after cell seeding.

### 4.3. Anti-Inflammatory Assay

To examine atherosclerotic signaling proteins, RAW 264.7 cells were seeded in 10-cm dishes at a density of 1 × 10^5^ cells. Inflammation in macrophages was induced by incubating them for 8 or 16 h in a medium containing LPS (0.01 μg/mL) without marine compound. For the anti-inflammatory activity assay, marine compound was added to the cells 5 min before the LPS challenge. After exposure to the test compounds, the cells were washed with ice-cold phosphate-buffered saline (PBS), lysed in ice-cold lysis buffer (50 mM Tris, pH 7.5, 150 mM NaCl, 1% Triton X-100, 100 μg/mL phenylmethylsulfonyl fluoride, and 1 μg/mL aprotinin), and centrifuged at 20,000× *g* for 30 min at 4 °C. The supernatants were decanted and reserved for Western blotting. Protein concentrations were measured using the DC protein assay kit (Bio-Rad, Hercules, CA, USA) using a method modified from that of Lowry *et al*. (1951) [54].

### 4.4. Western Blot Analysis

Western blotting was performed according to the method described in our previous study [55]. The sample was diluted with an equal volume of sample buffer (2% 2-mercaptoethanol, 2% sodium dodecyl sulfate (SDS), 0.1% bromophenol blue, 10% glycerol, and 50 mM Tris-HCl (pH 7.2)), and the protein lysate was loaded onto a 10% SDS-polyacrylamide gel. Electrophoresis was carried out at 150 V for 90 min. After electrophoresis, proteins were transferred onto a polyvinylidene difluoride membrane (PVDF; Immobilon-P, Millipore Corp., Billerica, MA, USA. (0.45-μm pore size)) overnight at 4 °C in transfer buffer (380 mM glycine, 50 mM Tris-HCl, 1% SDS, and 20% methanol). PVDF membranes were blocked with 5% non-fat dry milk in Tris-buffered saline containing 0.1% Tween (20 mM Tris-HCl, 0.1% Tween 20, and 137 mM NaCl (pH 7.4)) and then incubated overnight at 4 °C with primary antibodies. The primary iNOS, COX-2 and β-actin antibodies were used. A horseradish peroxidase-conjugated secondary antibody was used for detection. The bound antibody was detected by chemiluminescence (Millipore Corp., Billerica, MA, USA). Images were obtained using the UVP BioChemi Imaging System, and LabWorks 4.0 software (UVP, Upland, CA, USA) was used for relative densitometric quantification.

### 4.5. Lipid Droplets Fluorescence Assay

The lipid droplet fluorescence assay was carried out according to the manufacturer’s protocol. RAW 264.7 cells were seeded onto slides at a density of 1.2 × 10^5^ cells and cultured overnight in an incubator with a 5% CO_2_ and 95% air atmosphere. The following day, RAW 264.7 cells were treated with oleic acid (OA). Next, RAW 264.7 cells were treated with 10 μM WA-25 or simvastatin and then stimulated with LPS for 16 h. After the treatment, the cells were washed with Assay Buffer, fixed with Fixative Solution, and washed twice with Assay Buffer. Finally, Nile Red Staining Solution was used to detect lipid droplets. Fluorescence was visualized using a Leica DM6000 microscope (Leica Microsystems Inc., Buffalo Grove, IL, USA).

### 4.6. Immunocytochemistry

In order to examine atherosclerotic signaling proteins, RAW 264.7 cells were seeded onto slides at a density of 1.2 × 10^5^ cells. Immunocytochemistry was performed using modified methods as previously described [56]. After treatment, RAW 264.7 cells were fixed with 4% paraformaldehyde in PBS buffer for 8 min and then washed 3 times with PBS buffer. The cells were blocked with 4% normal goat serum dissolved in PBS containing 0.01% Triton X-100. After blocking, the RAW 264.7 cells were washed 3 times with PBS buffer and incubated overnight at 4 °C with primary antibodies (LAMP-1 or TGF-β1). Cells were washed 3 times with PBS buffer, blocked as described previously, and then incubated for 2 h at 37 °C with Alexa Fluor 488 (green fluorescence) or 594 (red fluorescence)-conjugated secondary antibody. Fluorescence was visualized using a Leica DM6000 microscope (Leica Microsystems Inc., Buffalo Grove, IL, USA).

### 4.7. Measurement of cAMP Levels

The cAMP concentrations were determined using a Cyclic AMP XP^®^ assay kit (Cell Signaling Technology, Inc., Beverly, MA, USA) according to the manufacturer's protocol. Briefly, Raw 264.7 cells were seeded at a density of 6 × 10^3^ cells/well in 96-well plate and incubated overnight. Following the exposure to the test compounds, the cells were washed with ice-cold PBS and lysed with ice-cold 1× lysis buffer. The HRP-linked cAMP solution was added to the assay plate, which was incubated at room temperature for 3 h, followed by 4 washes with the 1× wash buffer. Finally, the TMB substrate was added, and the absorbance measured at 450 nm. The cAMP content of the sample was calculated using a cAMP standard curve.

### 4.8. LDL Uptake Cell-Based Assay

The LDL uptake cell-based assay was performed according to the manufacturer’s protocol. RAW 264.7 cells were seeded onto slides at a density of 1.2 × 10^5^ cells and cultured overnight in an incubator under a 5% CO_2_ and 95% air atmosphere. The following day, the cells were treated with 10 μM WA-25 and then stimulated with LPS for 16 h. At the end of the treatment period, LDL conjugated to DyLight™ 549 was added into the culture medium for 4 h. Finally, fluorescence was visualized using a Leica DM6000 microscope (Leica Microsystems Inc., Buffalo Grove, IL, USA).

### 4.9. Data and Statistical Analysis

All data are presented as the mean ± standard error of the mean (SEM). For immunoblot and immunostaining analysis, the intensity of the experimental results was determined as the integrated optical density and compared to the average optical density of the corresponding control or LPS group. All data were analyzed by analysis of variance (ANOVA) with the Student-Newman-Keuls *post-hoc* test for multiple comparisons. Differences were significant when *p* was less than 0.05.

## 5. Conclusions

The present study demonstrates that WA-25 inhibits expression of two pro-inflammatory proteins, iNOS and COX-2, in LPS-stimulated RAW 264.7 macrophage cells. Although simvastatin inhibited iNOS expression, it up-regulated COX-2, and this effect may underlie some of the dangerous side effects of simvastatin. Moreover, WA-25 inhibited LPS-induced lipid droplet formation. Therefore, WA-25 simultaneously inhibited inflammation and prevented foam cell formation. Our study also shows that TGF-β1 could regulate inflammation and lipid metabolism in macrophages. Besides, WA-25 could improve the lipolysis instead of change the export of lipid. However, further studies will be required to determine the molecular mechanisms of WA-25 in more detail. Taken together, our results show that WA-25 may be a promising approach for the treatment of atherosclerosis that has fewer side effects than simvastatin.
